# Assessing endgame strategies for the elimination of lymphatic filariasis: A model-based evaluation of the impact of DEC-medicated salt

**DOI:** 10.1038/s41598-017-07782-9

**Published:** 2017-08-07

**Authors:** Morgan E. Smith, Brajendra K. Singh, Edwin Michael

**Affiliations:** Department of Biological Sciences, University of Notre Dame, Notre Dame, IN 46556 USA

## Abstract

Concern is growing regarding the prospects of achieving the global elimination of lymphatic filariasis (LF) by 2020. Apart from operational difficulties, evidence is emerging which points to unique challenges that could confound achieving LF elimination as extinction targets draw near. Diethylcarbamazine (DEC)-medicated salt may overcome these complex challenges posed by the endgame phase of parasite elimination. We calibrated LF transmission models using Bayesian data-model assimilation techniques to baseline and follow-up infection data from 11 communities that underwent DEC salt medication. The fitted models were used to assess the utility of DEC salt treatment for achieving LF elimination, in comparison with other current and proposed drug regimens, during the endgame phase. DEC-medicated salt consistently reduced microfilaria (mf) prevalence from 1% mf to site-specific elimination thresholds more quickly than the other investigated treatments. The application of DEC salt generally required less than one year to achieve site-specific LF elimination, while annual and biannual MDA options required significantly longer durations to achieve the same task. The use of DEC-medicated salt also lowered between-site variance in extinction timelines, especially when combined with vector control. These results indicate that the implementation of DEC-medicated salt, where feasible, can overcome endgame challenges facing LF elimination programs.

## Introduction

In 1997, the World Health Assembly (WHA) adopted resolution WHA 50.29 calling for the elimination of the mosquito-borne macroparasitic disease, lymphatic filariasis (LF), as a global public health problem. This lead to the establishment of the Global Program to Eliminate Lymphatic Filariasis (GPELF) by the World Health Organization (WHO) in 2000, which advocated the meeting of this goal by 2020 by all endemic countries through the application of annual mass drug administration (MDA) maintained over at least 4–6 years^1^. Despite the impressive expansion of the program since its inception in 2000, with 63 LF endemic countries currently under MDA^2^, delays in program implementation, issues with compliance, and contraindications of the two major drugs used in annual MDA, *viz*. ivermectin (IVM) and diethylcarbamazine (DEC), in some areas of Sub-Saharan Africa, suggest that the global elimination of LF is unlikely to be achieved by 2020 without rapid scale-up and/or via the discovery and application of more effective intervention technologies^3–5^.

Evidence, in the meantime, is also emerging in countries that have already received multiple annual MDAs, which points to the unique challenges that could confound achieving LF elimination as parasite extinction targets near. First, post-MDA surveillance studies reporting persistent microfilarial (mf) infections after many years of treatment efforts draw attention to the shifting dynamics of LF near transmission thresholds. For instance, three decades of treatment on the remote island of French Polynesia was not able to eradicate LF^6^, and cases in southern India, Nigeria, and Tanzania report that 8–10 years of annual MDA treatments have not halted transmission^7–9^. These slowed down but persistent low-level LF transmission regimes near elimination points could reflect the emergent effects of a range of factors, such as (1) the operation of diverse density dependent feedback loops that can maintain transmission resilience in the face of perturbations^4, 10^, (2) the impacts of diversity and modularity in the transmission process by involving two hosts and heterogeneous mosquito larval habitats that can contain the effects of perturbations locally, and (3) the inter-connectedness of host and vector populations over space and time that can sustain regional transmission even when local parasite extinction in individual sites is achieved^10–14^. In addition to these ecological shifts, operational challenges affecting control programs can also be amplified during the late stages of elimination. For example, achieving effective coverage in hard-to-reach communities, managing costs of surveillance at low infection levels, and preserving the commitment of international and local organizations become increasingly critical^15, 16^. With less than three years remaining for the GPELF to meet its 2020 target, these findings indicate that it is crucial not only to scale up programs and focus on developing new tools to accelerate the meeting of elimination targets, such as is being investigated with triple drug therapy^5, 17^, but also to update program strategies to appropriately manage these complex, emergent challenges that are uniquely posed by the endgame phase of parasite elimination^15^.

DEC-medicated salt has played a major role in the elimination of LF in a limited number of settings in Africa, Central America, and Asia^18–25^. Perhaps most famously, DEC-medicated salt was used in the vast majority of endemic regions of China starting in the 1980s after the first pilot trials in Tengxian County, Shandong Province, yielded impressive results. Following three years of treating positive cases with DEC tablets, elimination of LF was expedited by adopting community-based DEC-medicated salt treatment for six months. Community mf prevalence was reduced from 8.89% to 0.63% during the salt treatment and no new infections emerged during the next 10 years^26, 27^. Several additional trials followed subsequently in other provinces with similar results. Eventually comprising 73.9% of total treatments, DEC-medicated salt contributed considerably to the elimination of LF (marked by <1% mf prevalence) from all endemic provinces in China^26^.

However, because provinces in China adapted their strategies differently in response to evolving research, the DEC-medicated salt regimen used to control LF was not uniform^26, 28^. While generally medicated salt with 0.3% concentration DEC was administered for a period of time based on the mf rate and density reflected in surveys, the duration of treatment ranged from 3–6 months and the dosage ranged from 4.5 to over 9 grams DEC per person. Furthermore, in some areas, several intermittent 3-month courses of DEC-medicated salt were required to reduce infection. While salt was strictly controlled by health authorities in some regions, it was not feasible in others and was instead added to food products such as soy^26^. Finally, vector control and sanitation efforts were implemented in addition to chemotherapy in some regions^28^. This high variation between treatment regimens has made it difficult to extract a clear-cut strategy framework with respect to DEC-medicated salt from the success of the salt programs in China.

Here, we undertook a modelling study to systematically evaluate the utility of using DEC-medicated salt as an endgame strategy for achieving LF elimination by comparing its impact, relative to other current and proposed drug regimens, on the dynamics of parasite extinction during the endgame stage of elimination. The analysis was based on extending our recently developed dynamical LF transmission model to allow for the continuous application of DEC in the form of medicated salt^4, 29^. A Bayesian data-model assimilation approach was used to fit the model to longitudinal infection data observed in 11 study communities which underwent DEC salt medication, and the resulting calibrated models were used to assess the utility of using salt treatment for achieving LF elimination once infection was reduced to low levels. The results were also used to assess the impact of DEC medication as a strategy for addressing the challenge of heterogeneous LF transmission dynamics between sites during the elimination phase^4^. The performance of DEC-medicated salt as an endgame strategy in comparison with other drug regimens was also systematically evaluated in relation to variations in drug coverage and intensity of supplementary vector control (VC).

## Results

### Baseline mf prevalence data and site-specific LF models

**Figure 1:**
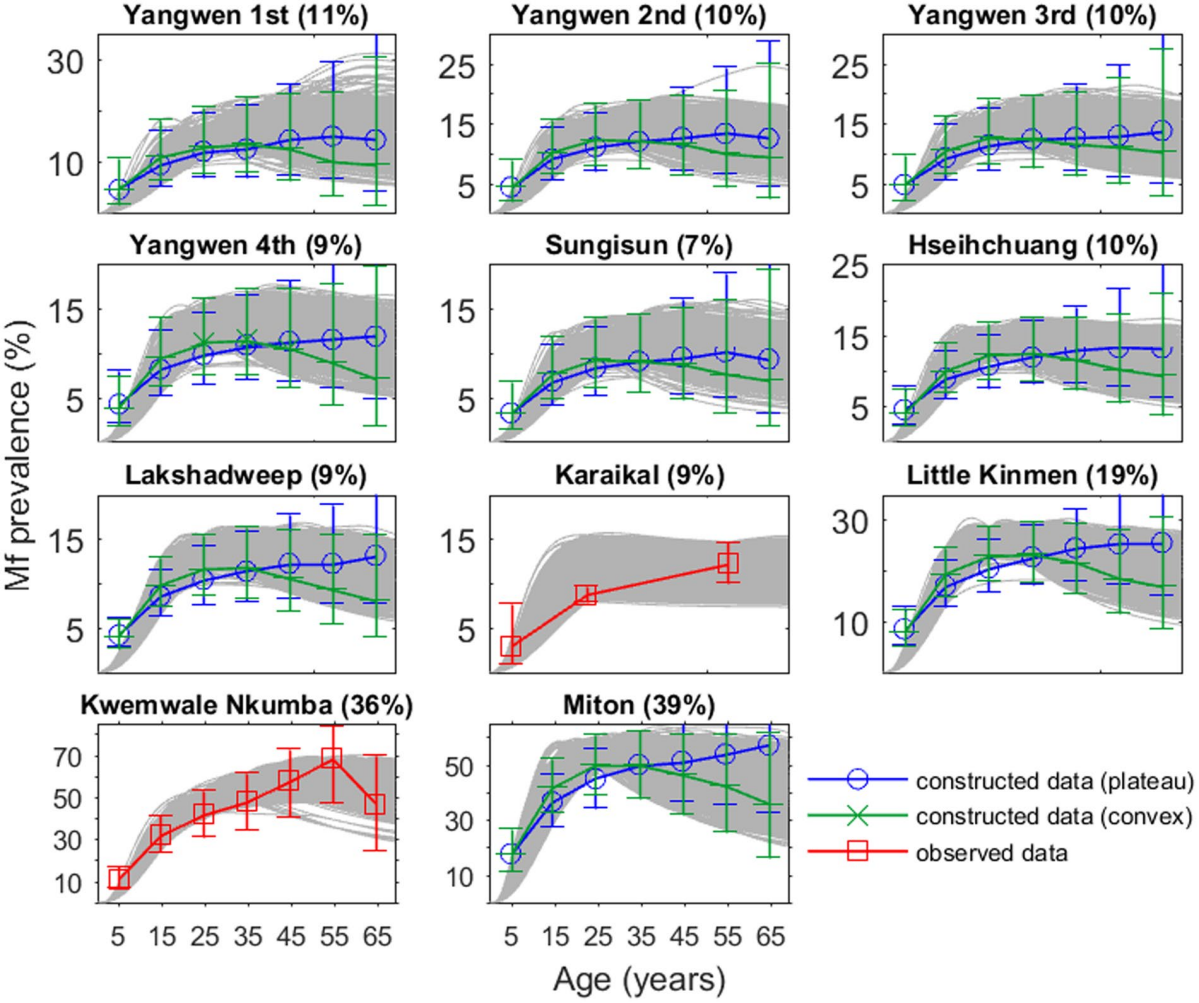
Model fits to baseline prevalence data for the 11 sites used in this study. Model fits generated by the BM fitting procedure are shown as gray age infection curves. The data shown is either observed age-stratified infection prevalence (red squares) or constructed age profiles from observed overall prevalence where age-stratified data was not available (blue circles and green crosses). The overall community level prevalence, corrected for blood volume, is noted in parentheses following the village names in each subplot.

The LF models (curves) identified for each study site based on their fits to baseline age-stratified mf prevalence data, either directly observed (squares) or constructed (circles and crosses), are shown in Fig. 1. As described in Methods, age-stratified mf prevalence data were constructed for 9 of the 11 study sites that provided only overall mf prevalence observations. All mf prevalence values were corrected to reflect sampling of 1 mL blood volumes using the transformation factors described previously^30^. The results show that the Bayesian Melding (BM)-based data assimilation fitting procedure which relied on the pass/fail binary fitting criterion was able to reproduce the baseline mf infection data for all 11 sites when compared to either the constructed or observed age prevalences (Fig. 1). Note that where age profiles have been constructed, an ensemble of fits to both plateau and convex age profiles was generated and used to describe the baseline age-infection prevalence in the corresponding field sites.

### Estimating the mf and worm killing rates of DEC-medicated salt

**Figure 2:**
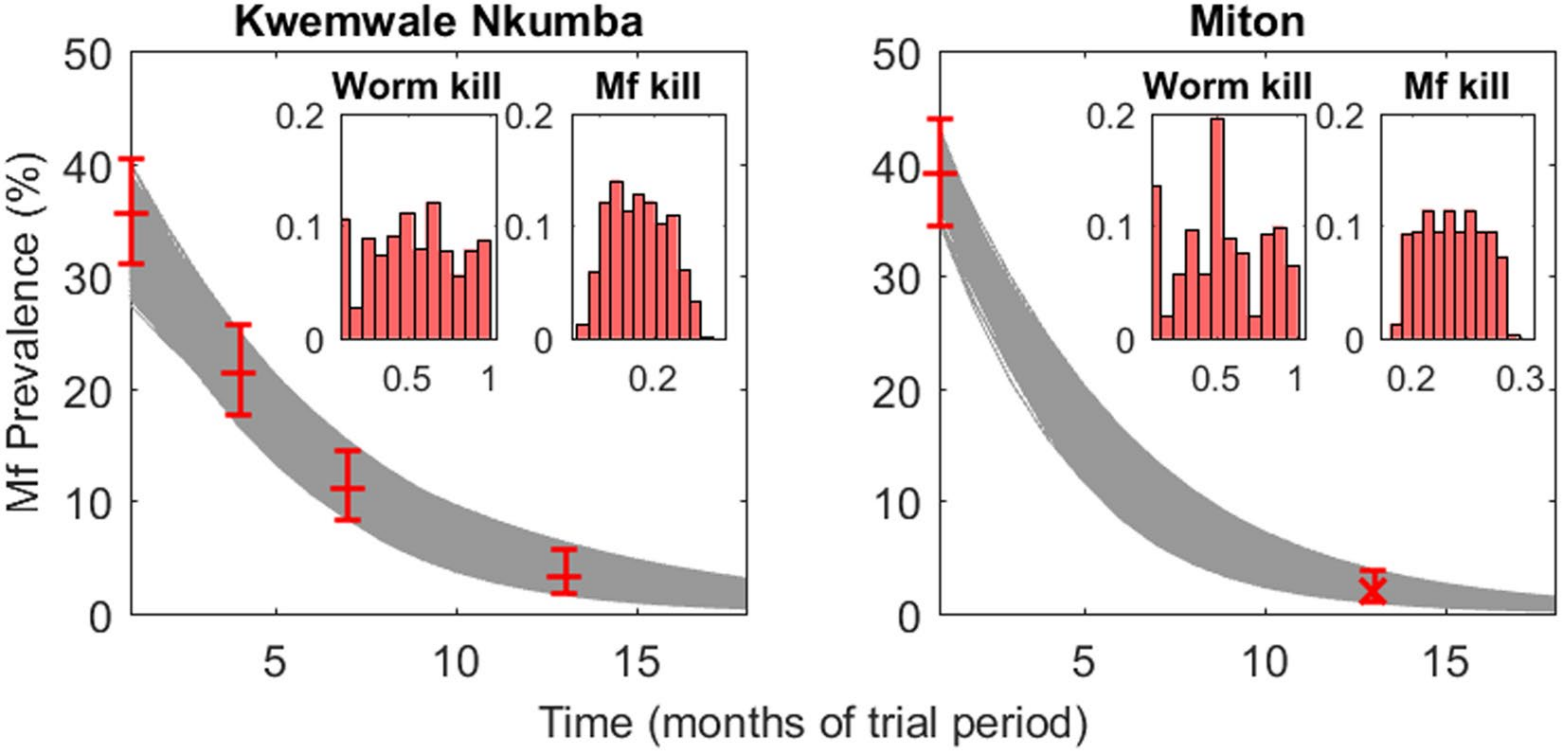
Estimates of site-specific drug efficacies calculated from model fits to intervention community trial data. The 500 parameter vectors fitted to baseline conditions were used to project the impact of DEC-medicated salt with each vector simulated 500 times via sampling from an initial range of plausible drug parameter values. Intervention survey data used to accept or reject a parameter vector are represented by red crosses with 95% binomial error bars in the main plot axes. Gray curves are the model fits which ultimately informed the monthly worm and mf kill rates. Inset plots show the posterior relative frequency distributions of the monthly worm and mf kill rates for each site.

For each of the 11 sites, the monthly worm kill rate, *ω*, and mf kill rate, *ε*, pertaining to DEC-medicated salt were estimated from their posterior parameter distributions derived from the parameter vectors that passed through both the baseline (observed/constructed) and follow-up overall mf prevalence data for each site. While there have been studies inferring the rate of mf clearance due to DEC-medicated salt^18–24, 31–33^, there have been limited corresponding studies inferring the kill rate of adult worms and they report discrepant results^33^. The posterior distributions of *ε* and *ω* for two of the study sites, Kwemwale/Nkumba, Tanzania, and Miton, Haiti, are shown in Fig. 2 as an example of the model-generated outcomes, with the posterior distributions for all sites given in Supplementary Figure S1. A Kruskal-Wallis test for equal distributions indicates that the posterior distributions of monthly worm and mf killing rates differred between the study sites (*p-values* < 2.2e–16), suggesting the existence of significant between-site heterogeneity in applied salt drug efficacies. The median values of estimated monthly worm and mf kill rates are tabulated for all villages in Supplementary Table S1, and indicate that these rates may range between 0.506 to 0.963 fractions of pre-patent and patent worms killed per month and 0.185 to 0.930 fractions of mf killed per month.

### Calculation of site-specific transmission endpoints

Distributions of model-generated mf breakpoints at the baseline annual biting rate (ABR) and at the threshold biting rate (TBR) were calculated for each site in this study using the numerical stability analysis method described previously^29^. The estimated between-site breakpoint distributions at both the ABR and at TBR statistically differed (significant Kruskal-Wallis test for equal distributions, *p-values* < 2.2 e–16). The 50%, 75%, and 95% elimination probability (EP) thresholds as derived via the application of an inverse cumulative density-based exceedance function to these site-specific breakpoint distributions are given in Supplementary Table S2 ^10, 34^. Typically, mf breakpoint values were lower at ABR (0.014% to 0.098% across sites) than at TBR (0.047% to 1.388%), and declined progressively with increasing EP probabilities (to as low as 0.014% at the 95% EP at ABR). In this study, the 95% EP threshold was considered the target endpoint when evaluating the effect of intervention strategies. Values of this threshold at ABR were used as targets for assessing parasite transmission elimination in the case of drug interventions, whereas values at TBR were used when the added effects of VC were modelled^4, 29^.

### Evaluating the impact of endgame intervention strategies

**Figure 3:**
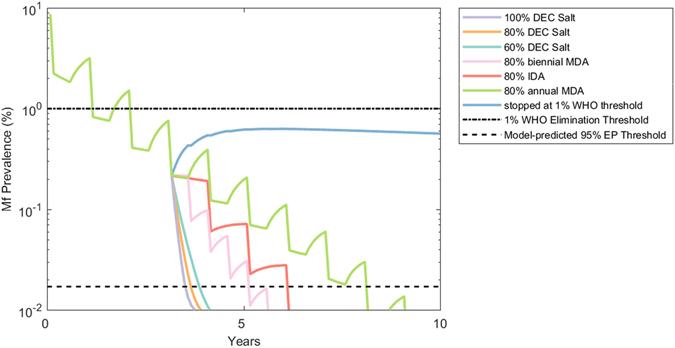
Decline in mf prevalence in Lakshadweep, India, resulting from several different intervention scenarios. The median model-predicted trajectory is shown for each intervention strategy with no background vector control. For the first four years in this simulation, annual MDA at 80% coverage is applied after which one of seven alternative treatment strategies is adopted. The WHO 1% mf threshold and the model-predicted 95% EP threshold are shown as horizontal lines. Note that the y-axis is given in log-scale to better visualize transitions to low prevalence values.

In this exercise, we evaluated and compared the impact of DEC salt as an endgame intervention strategy in relation to the performance of the other currently used or proposed LF drug interventions. An endgame strategy here refers to the use of a treatment regimen and/or VC only after annual MDA has decreased the community-level mf prevalence below the WHO-mandated threshold of 1% mf. Figure 3 plots this shift in intervention strategy after four rounds of annual MDA with DEC and albendazole (ALB) at 80% community coverage for Lakshadweep, India, showing the trajectories of seven alternative treatment options after crossing below the 1% mf prevalence threshold. The results show firstly that a significant reduction in the number of years of interventions can be achieved by switching to alternative treatment regimens as opposed to continuing with annual MDA. The most optimistic treatment option, however, is the application of DEC-medicated salt such that 100% community coverage is achieved, but, importantly, significant gains in intervention years saved were also achieved at lower coverages of 80% and 60% (Fig. 3). The trends seen in the case of Lakshadweep are echoed in the other study sites where DEC-medicated salt strategies consistently resulted in a quicker decline in mf prevalence over other treatment regimens (Table 1). Although varying between study sites, these results show that the application of DEC salt even at the low coverage of 60% generally needed under a year to achieve LF elimination in the investigated sites, while annual and biannual MDA options required significantly longer durations to accomplish the same task. Biannual MDA with DEC+ALB required up to 3 years and annual triple drug therapy with ivermectin, DEC, and ALB (IDA) needed up to 4 years on average to interrupt LF transmission, while annual DEC+ALB MDA required as high as around 6.5 years. The use of long-lasting insecticide nets (LLINs) in each site further reduced the number of years required to stop transmission during the elimination phase (Table 1). When vector control at 50% or 80% coverage was combined with drug treatments, salt intervention, regardless of drug coverage (60% to 100%), required only between 0.1 to 0.4 years to bring about parasite transmission interruption across the study sites in contrast to the annual MDA options (IDA and DEC+ALB regimens), which required between 1 to 5 years (Table 1). Biannual DEC+ALB MDA with VC required 0.5 to 1.9 years variously across sites to achieve this task, again demonstrating that among the options studied here, it was second to DEC salt medication in disrupting LF transmission during the elimination endgame phase (Table 1).

**Table 1:** Number of years of alternative interventions required to reach the site-specific 95% EP threshold after crossing the WHO 1% mf threshold.

VC Coverage	Drug Regimen (Coverage)	Yangwen 1st	Yangwen 2nd	Yangwen 3rd	Yangwen 4th	Sungisun	Hseihchuang	Lakshadweep	Karaikal	Little Kinmen	Kwemwale/Nkumba	Miton
VC 0%	DEC Salt (60%)	0.5	0.7	0.7	0.7	0.6	0.7	1.1	0.7	0.7	2.4	2.0
	DEC Salt (80%)	0.4	0.6	0.5	0.6	0.5	0.6	0.9	0.5	0.6	1.9	1.6
	DEC Salt (100%)	0.3	0.4	0.4	0.5	0.4	0.5	0.8	0.4	0.5	1.6	1.4
	Annual IDA (80%)	4.1	4.3	4.2	4.3	4.2	4.3	4.2	3.5	4.2	3.9	4.0
	Annual MDA (80%)	6.5	6.7	6.6	6.5	6.3	6.7	6.5	5.6	6.3	5.7	5.9
	Biannual MDA (80%)	2.8	3.0	2.8	2.9	2.8	2.9	2.9	2.3	2.9	2.7	2.8
VC 50%	DEC Salt (60%)	0.1	0.1	0.1	0.1	0.1	0.2	0.2	0.5	0.2	0.1	0.4
	DEC Salt (80%)	0.1	0.1	0.1	0.1	0.1	0.2	0.2	0.4	0.2	0.1	0.3
	DEC Salt (100%)	0.1	0.1	0.1	0.1	0.1	0.2	0.2	0.3	0.2	0.1	0.3
	Annual IDA (80%)	1.2	1.2	1.3	1.2	1.1	1.4	1.3	3.0	1.4	1.0	1.9
	Annual MDA (80%)	2.5	2.4	2.5	2.4	2.2	2.7	2.6	5.0	2.6	1.7	3.0
	Biannual MDA (80%)	0.8	0.8	0.8	0.7	0.6	0.9	0.8	1.9	0.9	0.5	1.0
VC 80%	DEC Salt (60%)	0.1	0.1	0.1	0.1	0.1	0.2	0.2	0.5	0.2	0.1	0.4
	DEC Salt (80%)	0.1	0.1	0.1	0.1	0.1	0.2	0.2	0.4	0.1	0.1	0.3
	DEC Salt (100%)	0.1	0.1	0.1	0.1	0.1	0.2	0.1	0.3	0.1	0.1	0.3
	Annual IDA (80%)	1.2	1.2	1.2	1.2	1.1	1.3	1.3	2.9	1.3	1.0	1.7
	Annual MDA (80%)	2.4	2.4	2.5	2.4	2.2	2.6	2.5	4.9	2.5	1.6	2.9
	Biannual MDA (80%)	0.8	0.7	0.8	0.7	0.6	0.9	0.8	1.9	0.8	0.5	1.0

The estimated mean number of years required to reduce mf prevalence to the 95% probability mf breakpoint threshold under each intervention in a particular site are reported.

**Figure 4:**
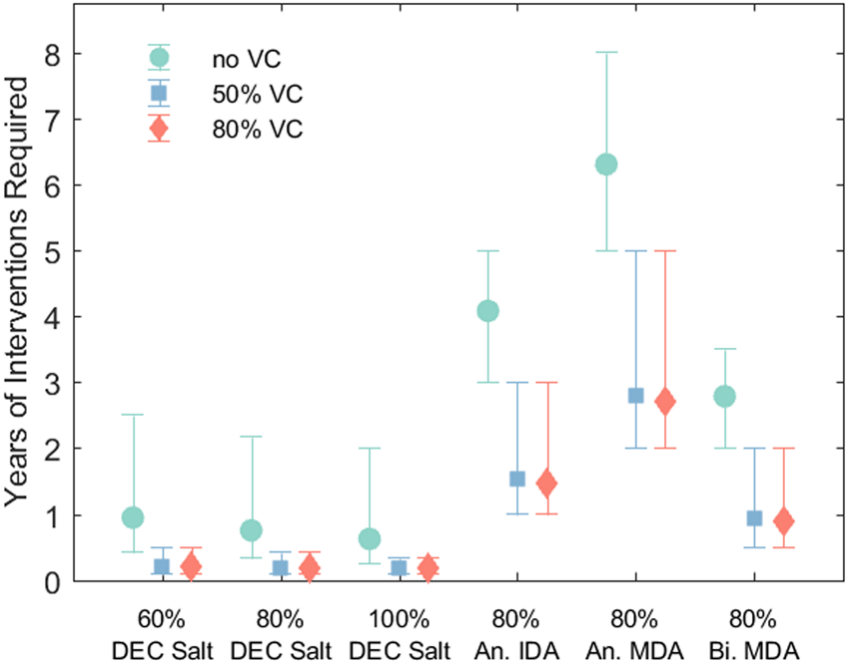
Number of years of alternative interventions required to reach site-specific 95% EP thresholds from 1% mf prevalence. Mean number of rounds across all study sites are shown as markers with 5^th^ and 95^th^ percentile error bars for each drug regimen and VC combination strategy.

Figure 4 graphically summarizes the differences in the impact of these strategies when the predicted timelines from 1% mf to the site-specific 95% EP threshold are aggregated across all sites. Results from a one-way ANOVA applied to these data indicated that the mean number of rounds or intervention years required under each of the 18 treatment strategies tested (6 drug regimens ×3 VC coverages) to achieve LF elimination in the present study sites were not equal (F(17,199404) = 77464, *p* < 0.0001). A post-hoc Tukey test confirmed the impression from Table 1 that combining DEC-medicated salt (60%, 80%, or 100% coverage) with VC (50% or 80% coverage) resulted overall in a statistically significant reduction in mean intervention time compared to all other treatment strategies for reducing mf prevalence from 1% to below the 95% EP threshold (*p* < 0.0001, Supplementary Table S3). Two sample F-tests for equality of variance furthermore indicated that the application of DEC-medicated salt (60%, 80% or 100% coverage) with VC (50% or 80% coverage) resulted in a significantly lower between-site variance in intervention times for achieving LF elimination compared to 80% IDA, annual MDA, or biannual MDA (*p* < 0.0001, Supplementary Table S4). By contrast, without VC, DEC-medicated salt at each of 60%, 80%, or 100% coverage had lower between-site variance in timelines to extinction than 80% annual MDA, while DEC-medicated salt only at 100% coverage produced lower variance in extinction timelines than 80% IDA (*p* < 0.001, Supplementary Table S4).

## Discussion

The results of this study show that applying DEC-medicated salt during the near-elimination phase when LF infection levels in endemic communities have been reduced to low levels may represent an efficient and robust endgame strategy for disrupting the transmission of this highly complex and geographically heterogeneous macroparasitic disease^4, 10, 29, 34, 35^. DEC-medicated salt was consistently shown in this modelling study, where a Bayesian statistical approach was used to calibrate and learn LF process-based mechanistic models based on data from diverse sites (Table 2 and 3), to reduce community mf prevalence more quickly than other status quo or newly proposed LF intervention strategies regardless of the operation of significant between-site transmission variability. The results show that adopting the use of DEC-medicated salt after reaching the WHO threshold by other means could lead to saving an average of 5.7 years of treatment across the present sites over continuing with annual MDA with DEC+ALB. More modest, yet still significant, gains are also made over annual IDA or even biannual DEC+ALB treatments.

**Table 2:** Baseline survey data for the 11 study sites.

Country	Village	Dominant Vector Species		Pre-treatment mf survey				Age-stratified survey data?	Source
			Survey Year	Blood sample volume (μL)	No. Sampled	No. mf Positive^a^	Mf prevalence (%)		
China	Yangwen 1^st^	*Culex pipiens pallens*	1973	60	543	58	10.7	no	22
China	Yangwen 2^nd^	*C.p. pallens*	1973	60	820	81	9.9	no	22
China	Yangwen 3^rd^	*C.p. pallens*	1973	60	749	76	10.1	no	22
China	Yangwen 4^th^	*C.p. pallens*	1973	60	1076	97	9.0	no	22
China	Sungisun	*C.p. pallens*	1973	60	1088	80	7.4	no	22
China	Hseihchuang	*C.p. pallens*	1973	60	1350	132	9.8	no	22
India	Lakshadweep	*C.p. fatigans*	1976	20	8361	726	8.7	no	23
India	Karaikal	*C. quinquefasciatus*	1981	20	14963	1323	8.8	yes	21, 24
Taiwan	Little Kinmen	*C. quinquefasciatus*	1970–1973	20	4794	896	18.7	no	18
Tanzania	Kwemwale/Nkumba	*C. quinquefasciatus* ^b^	1992	100	467	167	35.8	yes	20, 25, 57
Haiti	Miton	*C. quinquefasciatus*	1998^c^	20	409	160	39.1	no	19

^a^The number of positive cases is corrected for blood sample volume using a correction factor of 1.15 for 100 μL and 60 μL samples and 1.95 for 20 μL samples^30^.

^b^There is some uncertainty regarding the dominant vector species in Kwemwale/Nkumba, Tanzania. In addition to *C. quinquefasciatus*, both *Anopheles gambaie s.l*. and *An.funestus* have been cited as vectors responsible for transmission around Tanga and Muheza^57, 58^.

^c^The survey year was given by reference^59^.

**Table 3:** DEC-medicated salt community trial details. The ratio DEC by salt/DEC by MDA is a measure describing the total amount of DEC consumed per person by consuming medicated salt relative to the amount of DEC which would be consumed through the standard dose given for annual MDA interventions (6 mg/kg DEC plus 400 mg ALB) over the same time period of the DEC salt treatment.

Country	Village	DEC Salt Content (w/w)	Duration of Treatment (months)	Post-treatment mf survey^b^			Total DEC Dose (g)	Total DEC dose from equivalent MDA (g)	DEC by salt/DEC by MDA
				No. Sampled	No. mf Positive^a^	Mf prevalence (% reduction from baseline)			
China	Yangwen 1^st^	0.30%	6	557	5	0.9 (91.6)	7.5^c^	0.3	25
China	Yangwen 2^nd^	0.30%	6	866	10	1.2 (88.3)	7.5^c^	0.3	25
China	Yangwen 3^rd^	0.30%	6	755	10	1.3 (86.9)	7.5^c^	0.3	25
China	Yangwen 4^th^	0.30%	6	1103	13	1.2 (86.9)	7.5^c^	0.3	25
China	Sungisun	0.30%	6	1116	3	0.3 (96.4)	7.5^c^	0.3	25
China	Hseihchuang	0.30%	6	1369	20	1.5 (85.1)	7.5^c^	0.3	25
India	Lakshadweep	0.1–0.15%	27	12075	209	1.7 (80.1)	12	0.9	13.3
India	Karaikal	0.1–0.2%	48	1430	4	0.3 (96.8)	13.3	1.2	11.1
Taiwan	Little Kinmen	0.33%	6	5694	0	0.0 (100)	7.7	0.3	25.8
Tanzania	Kwemwale/Nkumba	0.33%	12	369	12	3.3 (90.9)	7.9^c^	0.3	26.3
Haiti	Miton	0.25%	12	409	8	2.0 (95.0)	17.4^c^	0.3	57.8

^a^The number of positive cases is corrected for blood sample volume using a correction factor of 1.15 for 100 μL and 60 μL samples and 1.95 for 20 μL samples30.

^b^Post-treatment mf prevalence observations were made at the end of the community trial with the exception of Little Kinmen for which data was available 1 year after the end of trial.

^c^Estimates were made assuming an average weight of 50 kg.

A number of studies and literature reviews addressing the efficacy of DEC-medicated salt corroborate our findings here of the rapidity by which this regimen may reduce LF transmission, citing for example that mf prevalence reductions are seen in 1–2 years with medicated salt which are typically only seen over 4–6 years under annual MDA^36, 37^. In the same vein, community trials from Tanzania point to how DEC-medicated salt can reduce mf prevalence much more efficiently than other strategies using DEC, and a comprehensive review found the regimen to be one the most effective means of clearing mf^20, 32, 38^. The modelling findings presented here, taken together with what has been documented empirically from the field, thus make a strong case for including DEC-medicated salt in LF elimination programs wherever feasible, especially as endemic communities begin to move toward the endgame phase of parasite elimination.

The results indicate that there may be two major reasons why DEC-medicated salt could be a particularly potent intervention for disrupting LF transmission during the endgame phase. First, the continuous consumption of the drug, even at low daily per capita dosages, leads to a cumulative impact on the survival of worms and mf which is shown to be much higher than that afforded by the higher-dosed annual or biannual MDA treatment (Table 3). This is an important outcome as it suggests that this intervention could serve as a robust measure to overcome the observed slowing down of LF system dynamics as infection nears critical breakpoint thresholds, which causes the system to “stick” around these regions and take a long time to either fall to the extinct state or rebound to its original endemic level^10^. The major and new insight from this work is that, in this situation, it may require the much more frequent drug pulses, such as that afforded by DEC-medicated salt, to break the resistance of the parasite system toward shifting to the extinct state once it enters the near extinction phase. The results indicate that DEC-medicated salt interventions can maintain this characteristic through low, medium, and high coverage scenarios, with even salt interventions applied at 60% coverage offering a more aggressive impact than MDA regimens provided at higher coverage levels (Table 1). With such promising efficiency, its use as an endgame strategy may also offer an effective means to overcome the coverage challenges of implementing a long duration community treatment program. Low rates of treatment coverage and compliance constitute one of the most highly cited reasons for program failure; this makes a regimen, such as DEC-medicated salt, which is forgiving of low coverage quite attractive.

The second major contribution of salt medication is highlighted by the results displayed in Fig. 4, which demonstrate how this intervention may overcome another emergent challenge during the endgame phase of parasite elimination programs, *viz*. the impact of spatial heterogeneities in transmission dynamics and their variable impact on timelines to extinction^4^. High uncertainty and complexity surrounding site-specific breakpoints presents a major challenge to country level program managers^39^, but concern is now also growing in connection with the impact that the emergence of hotspots of persistent transmission both within and between villages under control could have on achieving parasite elimination^14^. The modelling results presented here suggest that the variation in intervention requirements needed to overcome this heterogeneity is greatly reduced by the use of DEC-medicated salt compared to other intervention strategies, especially when coupled with VC (Fig. 4). This characteristic simplifies program design and management at the endgame phase by allowing the use of a single blanket approach across all endemic settings.

This study further underlines our previous findings regarding the added benefit of including VC for reducing the required period of interventions for achieving LF elimination under a drug treatment program^4, 29, 35, 40^. Here, however, we show that the use of LLINs even at 50% coverage in combination with DEC medicated salt can reduce the need for annual MDA interventions during the endgame phase by up to 6 years (Table 1). This was the case also when compared to annual IDA and biannual DEC+ALB MDA, where the corresponding saving in intervention durations were to the tune of 4 and 2 years, respectively (Table 1). In the most ideal case scenario, we predict that it may be possible to achieve site-specific elimination thresholds across communities in less than two additional months after reaching 1% mf prevalence by adopting a rigorous endgame intervention strategy combining VC (at least 50% coverage) with DEC-medicated salt administered at 100% coverage. This was the case even under a more practically achievable 60% and 80% salt coverage scenarios. These results support conclusions from other elimination programs of the importance of moving to the implementation of more intensified and diversified efforts during the endgame phase for affecting parasite elimination effectively^41^. It also supports the recent revived interest in India for the combined use of DEC salt and VC as an endgame strategy for accelerating LF elimination in that country, highlighting its growing significance in the current policy setting^42–44^.

Although the present results underscore the potential of using DEC-medicated salt to eliminate LF rapidly during the endgame phase, it is clear that there are implementation challenges associated with the approach that need to be considered if the intervention is to be adopted as an endgame measure. For instance, cooperation from local salt producers, community acceptance and compliance, and costs associated with production and distribution of the medicated salt have all been cited as barriers to implementation^33, 45^. However, the modelling results presented here indicate that high coverage of DEC-medicated salt is not necessary for its effectiveness in disrupting LF transmission during the low infection endgame phase, suggesting that it would be a worthwhile strategy for consideration even if cooperation is not granted by all salt producers or community members. We also show, in this regard, that the number of years of repeated visits to communities to distribute treatments as required by MDA programs is significantly reduced by the use of DEC-medicated salt even at low-medium coverages, which would overcome community fatigue to the repeated treatment cycles of the former programs. The preparation of medicated salt can be done either in small scale batches by methods as simple as mixing by hand or, where appropriate, can be produced at larger scales with the cooperation of salt producers and by leveraging processes already in place for the iodization of salt^27, 33, 36, 37, 45^. In Lakshadweep, India, quality control proved to be a challenge with respect to DEC dosage because community members washed the salt before use, which significantly reduced drug dosage^23^. Such challenges can be overcome with technological advances such as field testing for DEC content and with strong community mobilization, such as was achieved in Miton, Haiti^19, 45^. Effective engagement with current drug donors could also be a means for managing the production of high quality DEC salt in this respect. Further advantages include significantly reducing adverse side effects, removing the issue of drug distribution timing, increasing the likelihood of achieving high population coverage, and reducing manpower and financial costs^26, 33, 36, 46^. These considerations coupled with the modelling results from this study highlighting the effectiveness of DEC-medicated salt as an endgame strategy suggest that the intervention should be seriously considered where contextually appropriate.

Our modelling results are most sensitive to the concentration of DEC used typically in salt interventions. The limited number of trials which have been published do not form a consensus regarding the concentration of DEC to use in the salt. Concentrations reported by the studies included in this analysis ranged from 0.1–0.33% w/w and per-capita DEC intake ranged from 7.5–17.4 g assuming the average weight of a person to be 50 kg (Table 3). This amounts to the intake of 11–57 times more DEC through medicated salt than through rounds of annual MDA with DEC+ALB over the same time period, and well over the WHO recommended dose of 72 mg/kg DEC. One study did, however, find a weak positive correlation between per capita DEC consumption and the reduction of mf, motivating further investigation into this aspect of the regimen^37^. Stronger consensus on this detail would also be valuable to program managers in their efforts to operate cost-efficiently. However, our modelling results indicating little difference in the outcomes of applying medicated salt at varying coverages suggest that using even the lower end of concentrations studied here would still make the intervention a highly effective endgame option in comparison with MDA strategies. Our results are also based on infection data from only 11 sites with *Culicine* transmitted LF. Thus, even though the Bayesian melding approach we used allowed us to identify locality-specific LF models based on these site-specific data, the resulting models may not have captured the full range of heterogeneity that might be observed in the field despite baseline infection prevalences ranging from 8.8% to 31.1% across the present study sites (Table 2). We also had to derive age-prevalence patterns for those study sites that provided only overall prevalence data using typical age-infection profiles observed in LF endemic communities^47^. The ensembles of best-fitting models constructed using these approaches may either inflate or reduce the estimated heterogeneity in the modelled responses to the drug interventions studied here. However, again the tightness of the response to DEC-medication, in terms of timelines to extinction (Table 1), quantified across the 11 sites despite variations in their estimated endpoint values (Supplementary Table S2) indicate that our results may be robust to these limitations of the data used in this study. Finally, although including Anopheline LF sites may have the effect of reducing the timelines to extinction across all the drug regimens, their inclusion is unlikely to markedly change the overall finding from this study, *viz*. that DEC medicated salt treatment would more rapidly disrupt LF infection near breakpoints and reduce between-site variation in intervention durations to achieve site-specific elimination^4^.

In summary, we have presented a simulation study rooted in field data which indicates that using DEC-medicated salt even at moderate coverage may offer a potent intervention option to disrupt LF transmission efficiently and rapidly during the endgame phase compared to currently used or proposed MDA-based drug administration approaches. Including VC will further enhance this differential effect of community salt treatment. Our results are robust to data limitations and to the transmission heterogeneities that may govern LF extinction dynamics in the field. Although these results indicate a high prognosis for the use of DEC salt as an effective endgame intervention for eliminating LF transmission, major barriers to its programmatic use exist and need to be sufficiently resolved if its potential is to be realized. As highlighted by Lammie *et al*.^48^, a key impediment to the programmatic use of this intervention is related to its production and distribution to populations at high enough coverage levels. Experiences from China, India, Guyana and Haiti have demonstrated that this may be overcome at the programmatic level using various approaches that are specific to a country’s established salt production or importation and distribution system. This may include production and distribution through public systems (as was the case in India^43, 49^ through the country’s public distribution system (PDS)) or via the introduction of DEC-fortified salt into a competitive market as is being done in the case of Guyana^48^ and Haiti^50^. This suggests that regional or country-specific research into the necessary regulatory environment, partnerships with salt producers and importers, social marketing, learning from national salt iodization programs, and expansion of the skills of public health managers to manage fortified salt distribution will be required if the strategy is to be effectively deployed^48^. The second major barrier is the use of the salt intervention in areas of Sub-Saharan Africa co-endemic with onchocerciasis^51^. The primary issue here is the contraindicated use of DEC in these areas due to risk of inducing Mazzotti reactions and adverse ocular events in persons positive for *Onchocerca volvulus* ^51^. While this may present a major issue, it is pertinent to note that the low dosage of DEC salt administered through fortified salt coupled with low prevalence of onchocerciasis (as is expected in many current co-endemic regions in Africa due to long-term ivermectin MDA) could lessen this problem enough to support its deployment. This rationale, for example, underlies the growing interest in conducting randomized control trials for assessing the potential value of using triple drug therapy with IDA in these areas^51^. These considerations, along with the modelling results presented here, firstly indicate a need for policy makers to re-appraise the use of DEC-medicated salt in LF control programs during the endgame phase, and secondly support conduction of urgent field and operations research into its possible deployment in settings where it is deemed to be programmatically feasible.

## Methods

### Data

The data sources used in this analysis were assembled from 11 sites within Asia, Africa, and the Americas, which had conducted community trials using DEC-medicated salt against LF^18–25^. The data comprised pre-treatment baseline infection prevalence in each community determined using blood surveys for mf prevalence prior to the introduction of DEC-medicated salt (Table 2), as well as the corresponding post-treatment prevalences following each intervention (Table 3). While the methods of salt distribution and quality control varied between the sites, the medicated salt was reportedly used by nearly 100% of the study population in each field trial^18–25^ with support and cooperation from local authorities, salt vendors, and community members. The trials were conducted for 6–48 months in duration with aggregate doses ranging from 7.5 g to 17.4 g of DEC consumed through cooking salt (Table 3).

### LF transmission model

The LF transmission model used in the present analysis has been previously described in full elsewhere^10, 29, 30, 34, 35, 40^. In brief, the population dynamics of filarial infection are represented as coupled differential equations describing the changes in five state variables pertaining to infection in both human and vector host populations. In the human host, the pre-patent and patent worm loads (*P* and *W*, respectively), microfilarial intensity (*M*), and a measure of acquired immunity *(I*) to the parasite moderated by the total worm load (*P* + *W* = *W*_*T*_) are formulated as partial differential equations over time (*t*) and host age (*a*). In the mosquito host, the burden of infective L3 stage larvae (*L*) is described by an ordinary differential equation. The model is genus-specific with respect to the uptake of microfilariae by the mosquito vector. In this analysis, the functional form for mosquitoes of the *Culex* genus was applied. The model parameters and functions are fully described in the Supplementary Information (SI) (Supplementary Table S5).

### Model calibration to site-specific infection data using Bayesian Melding

We used a data-model assimilation technique based on the Bayesian Melding (BM) algorithm to calibrate and identify localized LF transmission models based on the mf prevalence data observed in each of our study sites. This was done by “melding” the observed baseline mf data from each site with model-generated outputs in order to learn models describing the localized parasite transmission dynamics per site^4, 29, 34, 52^. The fitted models from each site were then used to quantify each quantity of interest to this study, *viz*. mf breakpoints, threshold biting rates, and drug efficacy rates^4, 34, 35^. Typically, in the past, we had used the Sampling Importance Resampling (SIR) algorithm in order to select *N* (typically *N* = 500) parameter vectors, *θ*, or models applicable to a site based on their likelihood for describing the observed local data^4, 29, 34^. However, as noted by Raftery and Bao, SIR may not perform well in all cases^53^. In the current application, for example, the SIR algorithm selected very few unique parameter vectors because there were a handful of such vectors with high likelihoods given the data. These parameter vectors were consequently sampled many times resulting in a sparsely populated posterior parameter space. Bayesian techniques which bypass the evaluation of likelihoods, such as the use of a binary criterion of fit or model selection via Approximate Bayesian Computation (ABC), offer alternative methods to judge a model simulation based on its ability to mimic observed features of data^54^. For this work, the binary criterion of fit (pass/fail) proposed by Spear and Hubbard^54^ was implemented to address this problem using the algorithm below, wherein model generated mf prevalences, *M*¸ were judged against the observed age-stratified mf prevalence data, *D*, and *N* parameter vectors were selected only when their predicted age-prevalences fell within the 95% confidence intervals of the age-stratified data for *j*-2 data points (where *j* is the number of age groups in a particular site for which there is an observed prevalence):
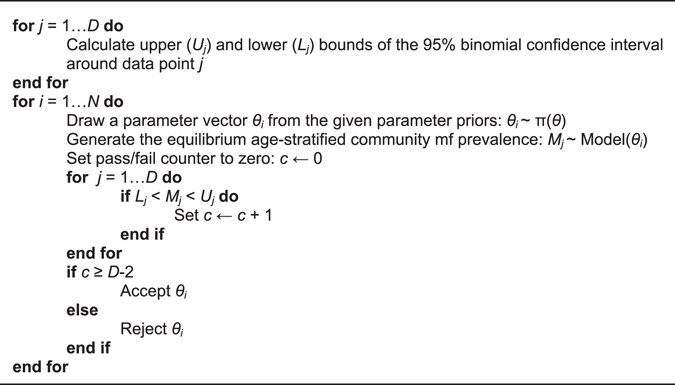


### Mf age profile construction in the absence of age-stratified infection data

The LF model BM fitting procedure relies on observed baseline age profiles of mf prevalence, but, in the present analysis, only overall community level mf prevalences were available for some sites. In this scenario, the observed overall prevalence was translated into theoretical age infection profiles using: 1) the national demographic profile applicable to the site in question, and 2) by conversion of the community-level mf prevalence to reflect either a plateau or concave age-infection profile known typically to occur in LF endemic regions^47^. The derived age-prevalence infection data were then used in the model fitting procedures described above, effectively allowing the integration of partially observed data into the model. See the SI for details (Supplementary Table S6 and S7, Supplementary Figure S2).

### Modelling the impact of DEC-medicated salt

The use of DEC-medicated salt as a treatment regimen differs from standard mass drug administration (MDA) primarily in that a small dose of DEC is consumed daily as opposed to a larger dose applied once or twice annually. In the case of annual or biannual MDA, the use of DEC is modelled by assuming an instantaneous killing effect on mf and worms at the time of ingestion. However, in the case of daily consumption, there is a continuous input of the drug at low concentration on a daily basis, suggesting a more frequent pulsing or spacing of discrete treatments than in the case of annual or biannual MDA. While it is possible to model DEC salt application using continuous functions, here, we approximated the daily drug intake instead as a monthly pulse where impacts on mf and adult worms are aggregated to reflect clearance rates per month. This was done to facilitate consistency with the monthly scale of the other parameters employed in the model^4, 29, 34^. Drug efficacy parameters quantified in this study thus reflected the monthly pre-patent and patent worm kill rate, *ω*, mf kill rate, *ε*, and a period of months, *p*, for which the newly produced mf continue to be killed after the *i* ^*th*^ administration of the drug at time *T*_*MDAi*_. These rates are applied in such a way that the total population burden of worms and mf are adjusted according to the observed drug coverage, *C* ^29^, where s is the worm sex ratio, α is the mf production rate, φ is the worm mating probability, and γ is the mf death rate (see Supplementary Table S5 for full parameter details).

1 $$\begin{array}{c}P(a,t+dt)=(1-\omega C)P(a,t)\\ W(a,t+dt)=(1-\omega C)W(a,t)\\ M(a,t+dt)=(1-\varepsilon C)M(a,t)\end{array}\}at\,time\,t={T}_{MD{A}_{i}}$$

2 $$\frac{\partial M(a,t)}{\partial t}+\frac{\partial M(a,t)}{\partial a}=(1-\varepsilon C)s\alpha \phi (W(a,t),k)W(a,t)-\gamma M(a,t),for\,{T}_{MD{A}_{i}} < t\le {T}_{MD{A}_{i}}+p$$

### Estimating drug parameter values

Site-specific monthly worm and mf kill rates as a result of DEC salt medication were estimated as follows. First, values of *ω* and *ε* were randomly selected from a range of plausible prior values using latin hypercube sampling and assigned to each of the model fits achieved using the BM procedure described above. This was repeated 500 times to derive a total set of 500 × 500 parameter vectors, each vector now containing its own quantified drug efficacy parameters. Second, simulations of the effects of monthly DEC salt treatment were run for each vector to obtain 500 mf prevalence trajectories forward in time per vector. Finally, all models whose mf trajectories arising from the monthly DEC salt intervention that passed through the 95% binomial confidence intervals of all but one follow-up survey data points in a site were selected, and their drug parameter values used to generate the corresponding site-specific distributions of values for *ω* and *ε*.

### Modelling the impact of MDA and vector control interventions

Two alternative combination MDA regimens; *viz*. 1) DEC+ALB, and 2) IDA, were modelled in this study to provide a comparison with the effects of DEC salt medication. The use of these drugs in MDA programs are modelled as described above with *T*_*MDAi*_ occurring annually or biannually with regimen-specific efficacy values^40^. To evaluate the added effect of VC as a supplement to drug-based interventions, the impact of long-lasting insecticidal bed nets with three main actions against mosquito biting was modelled: 1) deterring the insects from entering the home, 2) inhibiting their ability to feed on humans, and 3) killing them^55, 56^. The model formulation is described elsewhere^29^.

### Calculation of endpoints via numerical stability analysis

Site-specific transmission endpoints were calculated in this study as before using a numerical stability analysis approach^35^, which relies on a bisection root-finding method for identifying transmission and worm breakpoint thresholds^4, 29, 34^.

### Data and Code Availability

Custom code used to generate the results of this paper can be made available by the authors upon request. The authors declare that the data used in support of this study and its findings are available within the paper and its Supplementary Information files.

## Electronic supplementary material

Supplementary Information
