# Supplementary material for: Assessing endgame strategies for the elimination of lymphatic filariasis: A model-based evaluation of the impact of DEC-medicated salt
Source: Sci Rep. 2017 Aug 7;7:7386. doi: 10.1038/s41598-017-07782-9 (PMC5547057; doi:10.1038/s41598-017-07782-9)
Supplement: Supplementary file 1 — Supplementary Information [file 41598_2017_7782_MOESM1_ESM.pdf]

Supplementary Information

Assessing endgame strategies for the elimination of lymphatic filariasis: A model-based evaluation of the impact of DEC-medicated salt

Morgan E Smith[1], Brajendra K Singh[1], Edwin Michael[1]*

[1]Department of Biological Sciences, University of Notre Dame, Notre Dame, IN 46556, USA

*Corresponding author (emichael@nd.edu, 349 Galvin Life Science Center, University of Notre Dame, Notre Dame, IN 46556)

Supplementary Results

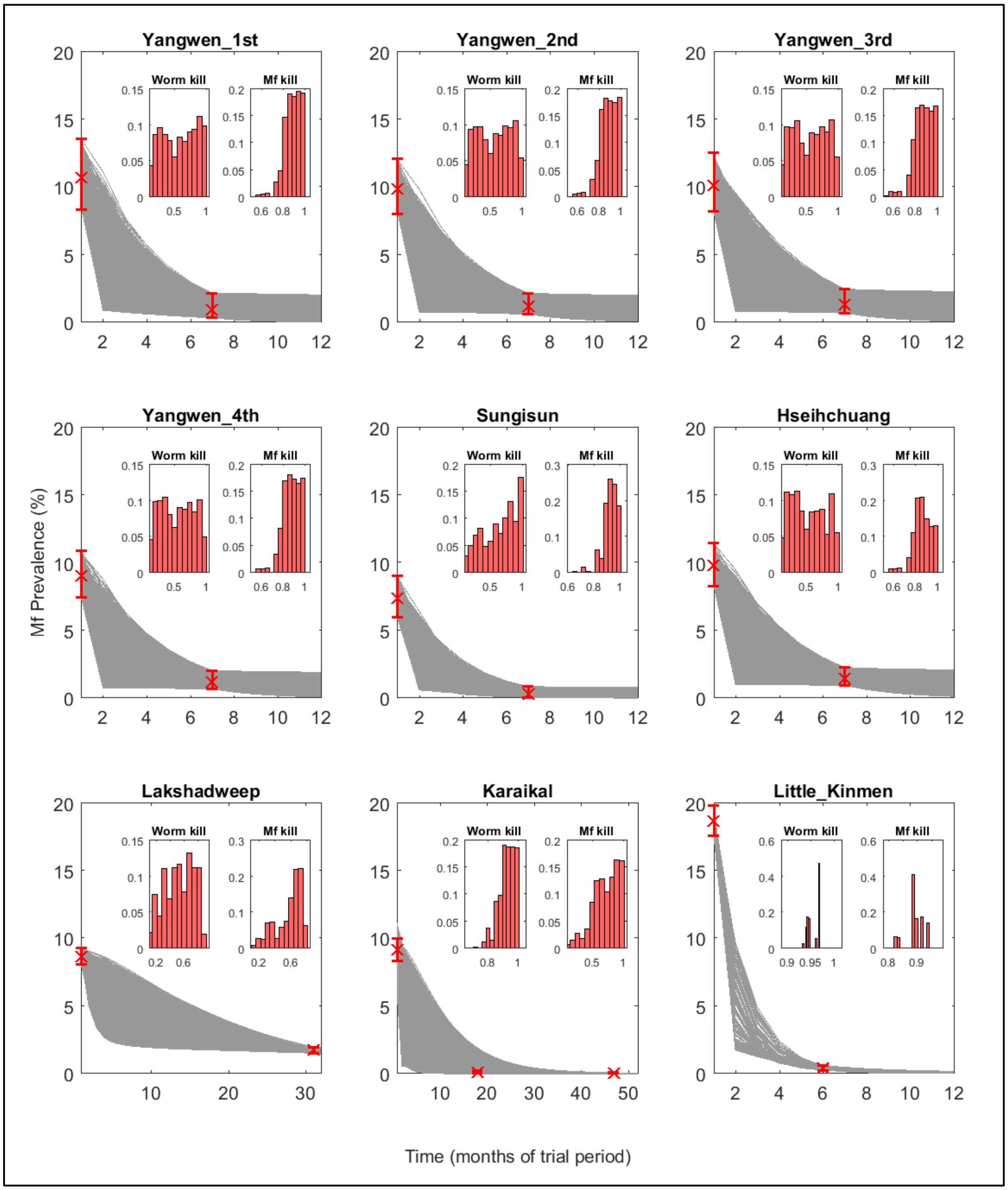

Supplementary Figure S1. Estimates of site-specific drug efficacies using model fits to intervention community trial data. This figure accompanies Figure 2 in the main text, reporting the results of the remaining trial sites. The 500 parameter vectors fitted to baseline conditions were used to project the impact of DEC-medicated salt with each vector simulated 500 times via sampling from an initial range of plausible drug parameter values. Intervention survey data used to accept or reject a parameter vector are represented by red crosses with 95% binomial error bars in the main plot axes. Gray curves are the model fits from which monthly worm and mf kill rates per site were calculated. Inset plots show the posterior relative frequency distributions of the monthly worm and mf kill rates for each site.

Supplementary Table S1. Median estimates of DEC-medicated salt monthly worm and mf killing rates as predicted by model fits to intervention community trial data.

Village	Worm Kill	Mf Kill
Yangwen 1st	0.590	0.895
Yangwen 2nd	0.567	0.888
Yangwen 3rd	0.564	0.876
Yangwen 4th	0.546	0.881
Sungisun	0.703	0.930
Hseihchuang	0.506	0.859
Lakshadweep	0.528	0.632
Karaikal	0.935	0.744
Little Kinmen	0.963	0.884
Kwemwale/Nkumba	0.539	0.185
Miton	0.507	0.236

Supplementary Table S2. Model-predicted site-specific mf breakpoints under ABR and TBR conditions for three elimination probabilities.

Village	At ABR			At TBR		
	50% EP	75% EP	95% EP	50% EP	75% EP	95% EP
Yangwen 1st	0.051	0.031	0.014	1.245	0.653	0.208
Yangwen 2nd	0.053	0.033	0.014	1.171	0.609	0.208
Yangwen 3rd	0.055	0.034	0.016	1.174	0.618	0.205
Yangwen 4th	0.055	0.034	0.016	1.338	0.659	0.199
Sungisun	0.051	0.034	0.015	1.388	0.703	0.209
Hseihchuang	0.055	0.034	0.016	1.303	0.671	0.186
Lakshadweep	0.058	0.037	0.017	1.179	0.538	0.179
Karaikal	0.098	0.067	0.038	0.168	0.112	0.047
Little Kinmen	0.063	0.040	0.019	1.098	0.586	0.181
Kwemwale/Nkumba	0.069	0.050	0.023	1.340	0.875	0.336
Miton	0.074	0.049	0.022	1.170	0.654	0.155

Supplementary Table S3. Mean differences between intervention times under Regimen A and Regimen B used in the post-hoc Tukey's HSD multiple comparison tests. Highlighted cells indicate there were significant differences between the group means being compared (p-values < 0.05).

			Regimen A																
			No VC					50% VC						80% VC					
			80% DEC	100% DEC	80% IDA	80% MDA	80% Biannual MDA	60% DEC	80% DEC	100% DEC	80% IDA	80% MDA	80% Biannual MDA	60% DEC	80% DEC	100% DEC	80% IDA	80% MDA	80% Biannual MDA
Regimen B	No VC	60% DEC	0.195	0.323	-3.137	-5.360	-1.841	0.736	0.759	0.772	-0.577	-1.851	0.017	0.739	0.761	0.773	-0.511	-1.772	0.054
		80% DEC		0.129	-3.331	-5.555	-2.036	0.541	0.565	0.578	-0.772	-2.045	-0.177	0.544	0.566	0.579	-0.706	-1.966	-0.141
		100% DEC			-3.460	-5.684	-2.165	0.413	0.436	0.449	-0.901	-2.174	-0.306	0.415	0.437	0.450	-0.834	-2.095	-0.269
		80% IDA				-2.224	1.295	3.873	3.896	3.909	2.559	1.286	3.154	3.875	3.897	3.910	2.626	1.365	3.191
		80% MDA					3.519	6.096	6.120	6.132	4.783	3.510	5.378	6.099	6.121	6.134	4.849	3.589	5.414
		80% Biannual MDA						2.577	2.601	2.613	1.264	-0.009	1.858	2.580	2.602	2.615	1.330	0.070	1.895
	50% VC	60% DEC							0.023	0.036	-1.313	-2.587	-0.719	0.003	0.025	0.037	-1.247	-2.508	-0.682
		80% DEC								0.013	-1.337	-2.610	-0.742	-0.020	0.001	0.014	-1.270	-2.531	-0.705
		100% DEC									-1.349	-2.623	-0.755	-0.033	-0.011	0.001	-1.283	-2.544	-0.718
		80% IDA										-1.273	0.594	1.316	1.338	1.351	0.066	-1.195	0.631
		80% MDA											1.868	2.590	2.611	2.624	1.340	0.079	1.905
		80% Biannual MDA												0.722	0.743	0.756	-0.528	-1.789	0.037
	80% VC	60% DEC													0.022	0.035	-1.250	-2.511	-0.685
		80% DEC														0.013	-1.272	-2.532	-0.707
		100% DEC															-1.284	-2.545	-0.719
		80% IDA																-1.261	0.565
		80% MDA																	1.826

Supplementary Table S4. Test statistics, degrees of freedom, and p-values for pairwise two sample F-tests testing the differences in variation of the time required to reach elimination under different treatment regimens. One-tailed two-sample F-tests were performed to test whether the variance of Regimen A (column label) was less than the variance of Regimen B (row label). Significant p-values are highlighted. In all comparisons, df1 = 11078 and df2 = 11078.

		Regimen A + no VC											
		60% DEC		80% DEC		100% DEC		80% IDA		80% MDA		80% Biannual MDA	
		F	p	F	p	F	p	F	p	F	p	F	p
Regimen B + no VC	60% DEC	1.000	0.500	0.813	0.000	0.750	0.000	0.797	0.000	2.349	1.000	0.584	0.000
	80% DEC	1.230	1.000	1.000	0.500	0.922	0.000	0.979	0.136	2.888	1.000	0.718	0.000
	100% DEC	1.333	1.000	1.085	1.000	1.000	0.500	1.062	0.999	3.132	1.000	0.779	0.000
	80% IDA	1.255	1.000	1.021	0.864	0.942	0.001	1.000	0.500	2.949	1.000	0.733	0.000
	80% MDA	0.426	0.000	0.346	0.000	0.319	0.000	0.339	0.000	1.000	0.500	0.249	0.000
	80% Biannual MDA	1.712	1.000	1.392	1.000	1.284	1.000	1.363	1.000	4.021	1.000	1.000	0.500
		Regimen A + 50% VC											
		60% DEC		80% DEC		100% DEC		80% IDA		80% MDA		80% Biannual MDA	
		F	p	F	p	F	p	F	p	F	p	F	p
Regimen B + 50% VC	60% DEC	1.000	0.500	0.675	0.000	0.528	0.000	16.960	1.000	34.410	1.000	7.365	1.000
	80% DEC	1.482	1.000	1.000	0.500	0.783	0.000	25.129	1.000	50.985	1.000	10.913	1.000
	100% DEC	1.893	1.000	1.278	1.000	1.000	0.500	32.108	1.000	65.145	1.000	13.944	1.000
	80% IDA	0.059	0.000	0.040	0.000	0.031	0.000	1.000	0.500	2.029	1.000	0.434	0.000
	80% MDA	0.029	0.000	0.020	0.000	0.015	0.000	0.493	0.000	1.000	0.500	0.214	0.000
	80% Biannual MDA	0.136	0.000	0.092	0.000	0.072	0.000	2.303	1.000	4.672	1.000	1.000	0.500
		Regimen A + 80% VC											
		60% DEC		80% DEC		100% DEC		80% IDA		80% MDA		80% Biannual MDA	
		F	p	F	p	F	p	F	p	F	p	F	p
Regimen B + 80% VC	60% DEC	1.000	0.500	0.710	0.000	0.574	0.000	14.776	1.000	31.630	1.000	6.797	1.000
	80% DEC	1.408	1.000	1.000	0.500	0.808	0.000	20.804	1.000	44.534	1.000	9.570	1.000
	100% DEC	1.742	1.000	1.238	1.000	1.000	0.500	25.746	1.000	55.114	1.000	11.844	1.000
	80% IDA	0.068	0.000	0.048	0.000	0.039	0.000	1.000	0.500	2.141	1.000	0.460	0.000
	80% MDA	0.032	0.000	0.022	0.000	0.018	0.000	0.467	0.000	1.000	0.500	0.215	0.000
	80% Biannual MDA	0.147	0.000	0.104	0.000	0.084	0.000	2.174	1.000	4.653	1.000	1.000	0.500

The mathematical model of LF transmission dynamics

We employed a *Culex* mosquito-vectored transmission model of LF to carry out the modelling work in this study[1-7]. Briefly, the state variables of this hybrid coupled partial differential and differential equation model vary over age (*a*) and/or time (*t*), representing changes in the pre-patent worm burden per human host $(P(a,t))$, adult worm burden per human host $(W(a,t))$, the microfilariae (mf) level in the human host modified to reflect infection detection in a 1 mL blood sample $(M(a,t))$, the average number of infective L3 larval stages per mosquito (*L*), and a measure of immunity $(I(a,t))$ developed by human hosts against L3 larvae. The state equations comprising this model are:

$$\frac{\partial P}{\partial t}+\frac{\partial P}{\partial a}=\lambda\frac{V}{H}h(a)\Omega(a,t)-\mu P(a,t)-\lambda\frac{V}{H}h(a)\Omega(a,t-\tau)\zeta$$
$$\frac{\partial W}{\partial t}+\frac{\partial W}{\partial a}=\lambda\frac{V}{H}h(a)\Omega(a,t-\tau)\zeta-\mu W(a,t)$$
$$\frac{\partial M}{\partial t}+\frac{\partial M}{\partial a}=\alpha s\phi[W(a,t),k]W(a,t)-\gamma M(a,t)$$
$$\frac{\partial I}{\partial t}+\frac{\partial I}{\partial a}=W_T(a,t)-\delta I(a,t)$$
$$\frac{dL}{dt}=\lambda\kappa g\int\pi(a)(1-f[M(a,t)])da-(\sigma+\lambda\psi_1)L$$
$$L^*=\frac{\lambda\kappa g\int\pi(a)(1-f[M(a,t)])da}{\sigma+\lambda\psi_1}$$

The above equations involve partial derivatives of four state variables (*P* - pre-patent worm load; *W* - adult worm load; *M* - microfilaria intensity; *I* - immunity to acquiring new infection due to the pre-existing total worm load where $W_T = W(a,t) + P(a,t)$). Given the faster time scale of infection dynamics in the vector compared to the human host, the infective L3-stage larval density in mosquito population is modelled by an ordinary differential equation essentially reflecting the significantly faster time-scale of the infection dynamics in the vector hosts. This allows us to make the simplifying assumption that the density of infective stage larvae in the vector population reaches a dynamic equilibrium (denoted by L^*) rapidly[1, 2, 5, 8, 9]. This basic coupled

immigration-death structure of the model as well as its recent extensions has been extensively discussed previously[1-3, 5, 8, 9]. The effects of worm patency are captured by considering that at any time t, human individuals of age less than or equal to the pre-patency period, τ, will have no adult worms or Mf, and the rate at which pre-patent worms survive to become adult worms in these individuals at $a > \tau$ is given by $\zeta = \exp(-\mu\tau)$. The term $f(M)$ enables us to account for the different establishment and development rates of the incoming L3-stage larvae as adult worms depending on the genus of mosquito vectors. For culicine-mediated LF, the functional form reflecting a negative-density dependent development of L3 larvae from ingested mf was deployed in the model[1]. See Supplementary Table S5 for the description of all the model parameters and functions.

Supplementary Table S5 - Description the basic LF model parameters and functions used in the model.

Parameter	Definition (*units*)	Range	Refs
λ	Number of bites per mosquito (*per month*)	[5, 15]	1, 2, 5, 10, 11
τ	Pre-patency period	[6, 9]	12
s	Proportion of female worms	0.5	-
μ	The worm mortality rate (*per month*)	[0.008, 0.018]	1, 2, 5, 13-16
α	Production rate of microfilariae per worm (*per month*)	[0.25, 1.5]	1, 2, 5, 17
γ	The death rate of the microfilariae (*per month*)	[0.08, 0.12]	1, 5, 15, 17
g	Proportion of mosquitoes which pick up infection when biting an infected host	[0.259, 0.481]	1, 5, 18
κ	Maximum level of L3 given Mf density	[3.955, 4.83]	1, 5
k_0	The basic location parameter of negative binomial distribution used in aggregation parameter ($k = k_0 + k_{Lin}M$)	[0.000036, 0.00077]	1, 5, 19, 20
δ	Immunity waning rate (*per month*)	[0, 0.000001]	1, 5
V/H	Ratio of number of vector to hosts	$MBR^{\#} / \lambda$	data
k_{Lin}	The linear rate of increase in the aggregation parameter defined above	[0.00000024, 0.282]	1, 5, 19, 20
σ	Death rate of mosquitoes (*per month*)	[1.5, 8.5]	1, 5, 20
ψ_1	Proportion of L3 leaving mosquito per bite	[0.12, 0.7]	17
ψ_2	The establishment rate[1]	[0.0000398, 0.00364]	1, 2, 5, 21
H_{Lin}	A threshold value used in $h(a)$ to adjust the rate at which individuals of age a are bitten: linear rise from 0 at age zero to 1 at age H_{lin} in years. $h(a) = a / H_{Lin}$ for $a < H_{Lin}; h(a) = 1$ for $a \geq H_{Lin}$	[12, 240] months	1, 5, 9
r	Gradient of Mf uptake[2]	[0.0495, 0.22]	1, 5
c	Strength of acquired immunity	[0.0000003, 0.0109]	1, 5
I_C	Strength of immunosuppression[3]	[0.5, 5.5]	1, 5
S_C	Slope of immunosuppression function[4] (*per worm/month*)	[0.01, 0.19]	1, 5
MDA drug-related parameters			
ω	Worm killing efficacy (instantaneous)	dependent on drug regimen	3
ε	Microfilariae killing efficacy (instantaneous)	dependent on drug regimen	3
δ_{reduc}	Reduction in the worm's fecundity over a period of time p	dependent on drug regimen	3
p	A time period during which the drug remains efficacious in reducing the fecundity of the surviving adult worms	dependent on drug regimen	3
C	Percentage of the population administered the drug	data	data
**Implementing vector control (VC)** such as IVM modifies the V/H ($= MBR/\lambda$)			
MBR_{VC}	$MBR_{VC} = MBR_0 \exp[a_1 t]$, with $a_1 < 0$ for $\forall t$ when VC is ON, otherwise $a_1 > 0$.	data and estimates	19, 20
**Description**	**Mathematical expressions of the functions**	**Parameters**	
Probability that an individual is	$\pi(a) = A_0 \exp[-B_0 a]$	Human age a in month, A_0 and B_0 estimated from country demographic	1, 5, 9

of age *a* $\pi(a)$		data	
Larvae establishment rate (modified by acquired immunity) $\Omega(a,t)$	$L^{*}\psi_1\psi_2 g_1(I) g_2(W_T)$	ψ_1 - proportion of L3 leaving mosquito per bite; ψ_2 - the establishment rate[1]	-
Adult worm mating probability $\phi(W,k)$	$1-\left(1+\frac{W}{2k}\right)^{-(1+k)}$	k – negative binomial aggregation parameter	[2, 5, 22]
Immunity to larval establishment $g_1(I)$	$\frac{1}{1+cI}$	c – strength of immunity to larval establishment	[1, 5]
Host immunosuppression $g_2(W_T)$	$\frac{1+I_C S_C W_T}{1+S_C W_T}$	I_C – strength of immunosuppression; S_C – slope of immunosuppression	[1, 5]

[1]The proportion of L3-stage larvae infecting human hosts that survive to develop into adult worms[2].
[2]The gradient of Mf uptake *r* is a measure of the initial increase in the infective L3 larvae uptake by vector as *M* increases from 0[2, 9].
[3] The facilitated establishment rate of adult worms due to parasite-induced immunosuppression in a heavily infected human host
[4] The initial rate of increase by which the strength of immunosuppression is achieved as *W* increases from 0[23].
Note MBR (monthly biting rate) serves as an input to initialize the model, measured as mosquito bites per person per month, the value of which may be obtained from entomological surveys conducted in study sites. In the absence of the observed MBR value, the model has been adapted to estimate it from the community-level Mf prevalence data.

Mf age profile construction in the absence of age-stratified infection data

The Bayesian Melding (BM) procedure for calibrating our deterministic LF model with data relies on baseline age profiles of microfilaria (mf) prevalence, but, in some cases, only the overall community level mf prevalence was available for a particular site in this study. This therefore required the translation of the overall prevalence into theoretical age infection profiles. This was done by firstly fitting equations to datasets (Supplementary Table S6 and Supplementary Table S7) of age-stratified mf infection data which qualitatively follow either plateau or convex profiles (Supplementary Figure S2). Specifically, plateau and convex age prevalence curves were defined by the following equations where P is the mf prevalence as a function of age a:

$P(a) = \frac{0.0018a}{1 + \frac{a}{11.99}}$ for plateau-type age profiles, and

$P(a) = 0.029a * \exp[-0.033a]$ for convex-style age profiles.

The number of infected individuals in each age class was then derived from the observed overall mf prevalence by applying these equations to the overall prevalence, while subdividing the total population into age-classes in each site according to their respective national age-demographic patterns.

Supplementary Table S6. Plateau-style age profile data

Site	Age	No. Examined	No. mf Positive	Reference
Quilandy Panchayath	< 1	94	0	24
	1-4	1056	30.624	
	5-14	3515	168.72	
	15-24	2451	154.413	
	25-34	1394	76.67	
	35-44	1060	76.32	
	44+	1535	96.705	
Lucknow, Sitapur, Hardoi (rural)	0-1	124	0	25
	2-5	526	4	
	6-10	775	18	
	11-20	1246	47	
	21-30	1784	72	
	31-40	710	45	
	41-50	399	22	
	50+	319	25	
Sitapur Town (urban)	0-1	45	0	25
	2-5	202	1	
	6-10	434	8	
	11-20	1076	57	
	21-30	1329	70	
	31-40	637	46	
	41-50	288	20	
	50+	169	13	
Greater Bombay	1-5	4806	43	26
	6-10	6208	143	
	11-20	10226	394	
	21-30	12744	503	
	31-40	11905	306	
	41-50	3353	158	
	50+	1963	82	
Ghazipur (Rural)	0-1	34	0	27
	2-5	619	5	
	6-10	1909	38	
	11-20	2973	192	
	21-30	2188	225	
	31-40	1670	198	
	41-50	1107	117	
	50+	712	78	

Gopalpur	0-4	336	7.918	28
	5-14	1438	138.944	
	15-24	973	99.01	
	25-34	553	59.918	
	35-44	534	53.128	
	45-54	407	43.999	
	55+	418	36	
Haldiapada	0-4	609	46.014	28
	5-14	1345	116.883	
	15-24	748	95.333	
	25-34	626	75.206	
	35-44	537	72.834	
	45-54	372	50.118	
	55+	427	50.035	

Supplementary Table S7. Convex style age profile data

Site	Age	No. Examined	No. mf Positive	Reference
Bhagalpur	0-5	345	26	29
	6-10	601	45	
	11-20	204	62	
	21-30	334	56	
	31-40	234	32	
	41-50	144	21	
	51+	78	10	
Gonda Town	0-1	13	0	30
	2-5	205	5	
	6-10	443	23	
	11-20	807	65	
	21-30	773	88	
	31-40	509	57	
	41-50	206	19	
	50+	142	14	
Pondicherry	0-5	121	4	31
	6-10	211	29	
	11-15	224	43	
	16-20	195	52	
	21-30	328	60	
	31-40	215	38	
	41-50	121	22	
	51-60	82	16	
	61+	52	12	
Gorakhpur	0-1	11	0	32
	2-5	1678	56	
	6-10	2867	236	
	11-20	4070	675	
	21-30	5212	778	
	31-40	3045	429	
	41-50	4051	590	
	50+	1400	180	
Chaliyum Ward I	1-4	283	0	33
	5-14	926	92	
	15-24	509	77	
	25-34	324	38	
	35-54	346	27	
	55+	158	9	

Howrah	0-4	32	0	34
	5-9	32	1.28	
	10-14	47	1.41	
	15-19	30	3.3	
	20-29	96	27.84	
	30-39	54	14.58	
	40-49	58	10.44	
	50+	51	11.73	

A.

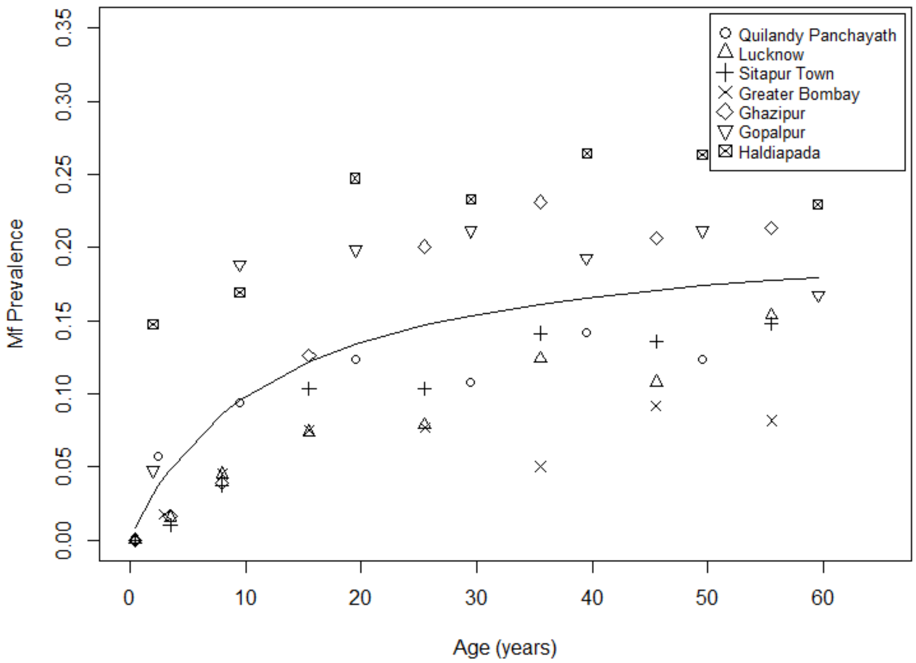

B.

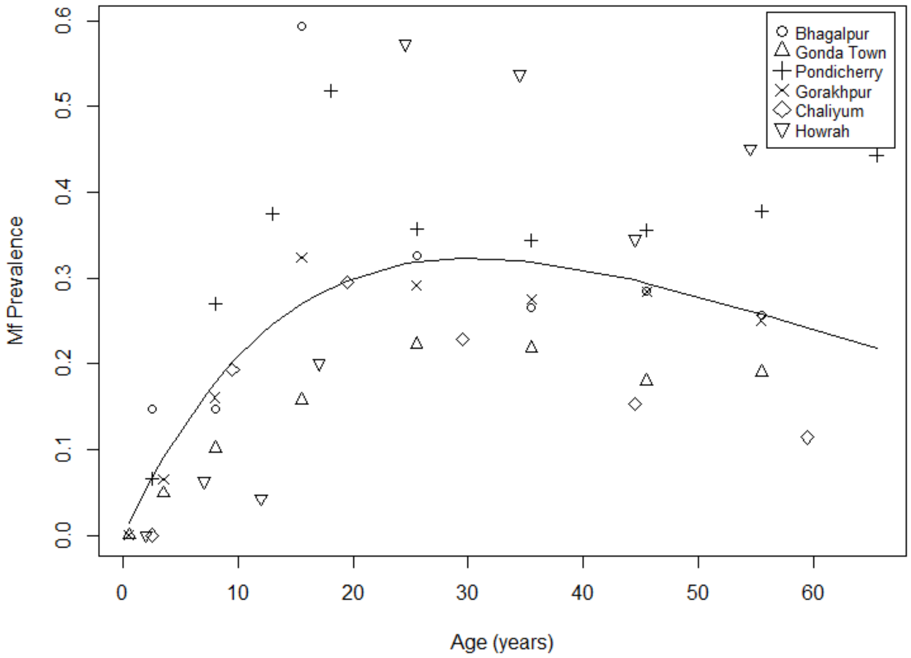

Supplementary Figure S2. Fits to (A) plateau- and (B) convex-style age infection profiles.

References

1. Gambhir, M. & Michael, E. Complex ecological dynamics and eradicability of the vector borne macroparasitic disease, lymphatic filariasis. *PLoS One* **3**, e2874 (2008).

2. Gambhir, M. *et al*. Geographic and ecologic heterogeneity in elimination thresholds for the major vector-borne helminthic disease, lymphatic filariasis. *BMC biology* **8**, 1 (2010).

3. Michael, E. *et al*. Mathematical modelling and the control of lymphatic filariasis. *The Lancet infectious diseases* **4**, 223-234 (2004).

4. Michael, E., Malecela-Lazaro, M. N., Kabali, C., Snow, L. C. & Kazura, J. W. Mathematical models and lymphatic filariasis control: endpoints and optimal interventions. *Trends Parasitol.* **22**, 226-233 (2006).

5. Singh BK and Bockarie MJ and Gambhir M and Siba PM and Tisch DJ and Kazura J and others. in *Sequential Modelling of the Effects of Mass Drug Treatments on Anopheline-Mediated Lymphatic Filariasis Infection in Papua New Guinea* (PLoS One, 2013).

6. Singh, B. K. & Michael, E. Bayesian calibration of simulation models for supporting management of the elimination of the macroparasitic disease, Lymphatic Filariasis. *Parasit vectors* **8**, 1-26 (2015).

7. Michael, E. & Singh, B. K. Heterogeneous dynamics, robustness/fragility trade-offs, and the eradication of the macroparasitic disease, lymphatic filariasis. *BMC medicine* **14**, 1 (2016).

8. Chan, M. S. *et al*. Epifil: a dynamic model of infection and disease in lymphatic filariasis. *Am. J. Trop. Med. Hyg.* **59**, 606-614 (1998).

9. Norman, R. *et al*. EPIFIL: the development of an age-structured model for describing the transmission dynamics and control of lymphatic filariasis. *Epidemiol. Infect.* **124**, 529-541 (2000).

10. Rajagopalan, P. Population dynamics of culex pipiens fatigans, the filariasis vector, in pondicherry: influence of climate and environment. *Proc Ind Nat Science Acad B* **46**, 745-752 (1980).

11. Subramanian, S., Manoharan, A., Ramaiah, K. D. & Das, P. K. Rates of acquisition and loss of Wuchereria bancrofti infection in Culex quinquefasciatus. *Am. J. Trop. Med. Hyg.* **51**, 244-249 (1994).

12. Scott, A. L. & Nutman, T. Lymphatic-dwelling filariae. *Lymphatic filariasis.*, 5-39 (2000).

13. Vanamail, P., Subramanian, S., Das, P. K., Pani, S. P. & Rajagopalan, P. K. Estimation of fecundic life span of Wuchereria bancrofti from longitudinal study of human infection in an endemic area of Pondicherry (south India). *Indian J. Med. Res.* **91**, 293-297 (1990).

14. Evans, D. B., Gelband, H. & Vlassoff, C. Social and economic factors and the control of lymphatic filariasis: a review. *Acta Trop.* **53**, 1-26 (1993).

15. Ottesen, E. & Ramachandran, C. Lymphatic filariasis infection and disease: control strategies. *Parasitology Today* **11**, 129-130 (1995).

16. Vanamail, P. *et al*. Estimation of the fecund life span of Wuchereria bancrofti in an endemic area. *Trans. R. Soc. Trop. Med. Hyg.* **90**, 119-121 (1996).

17. Hairston, N. G. & de Meillon, B. On the inefficiency of transmission of Wuchereria bancrofti from mosquito to human host. *Bull. World Health Organ.* **38**, 935-941 (1968).

18. Subramanian, S. *et al*. The relationship between microfilarial load in the human host and uptake and development of Wuchereria bancrofti microfilariae by Culex quinquefasciatus: a study under natural conditions. *Parasitology* **116**, 243-255 (1998).

19. Subramanian, S., Pani, S., Das, P. & Rajagopalan, P. Bancroftian filariasis in Pondicherry, south India: 2. Epidemiological evaluation of the effect of vector control. *Epidemiol. Infect.* **103**, 693-702 (1989).

20. Das, P. *et al*. Bancroftian filariasis in Pondicherry, south India–epidemiological impact of recovery of the vector population. *Epidemiol. Infect.* **108**, 483-493 (1992).

21. Ho, B. C. & Ewert, A. Experimental transmission of filarial larvae in relation to feeding behaviour of the mosquito vectors. *Trans. R. Soc. Trop. Med. Hyg.* **61**, 663-666 (1967).

22. May, R. M. Togetherness among schistosomes: its effects on the dynamics of the infection. *Math. Biosci.* **35**, 301-343 (1977).

23. Duerr, H., Dietz, K. & Eichner, M. Determinants of the eradicability of filarial infections: a conceptual approach. *Trends Parasitol.* **21**, 88-96 (2005).

24. Jain, D. C., Menon, P. K., Sethumadhavan, K. V., Johney, V. M. & Ghosh, T. K. Epidemiology of bancroftian filariasis in a semi-urban community of Kerala State. *J. Commun. Dis.* **21**, 265-271 (1989).

25. Singh, M., Rastogi, K., Singh, R. & Srivastava, V. Observations on rural filariasis in Sitapur District (Uttar Pradesh). *Indian J. Malariol.* **17**, 303-310. (1963).

26. Basu, P., Rao, V. & Pattanayak, S. Filariasis in greater Bombay-results of a rapid survey conducted in June, 1965. *Bull.Indian Soc.for Malaria & other Communicable Dis.* **4**, 296-317. (1967).

27. Chand, D., Singh, M. & Pathak, V. Filariasis in the District of Ghazipur (Uttar Pradesh). *Indian J. Malariol.* **15**, 21-29 (1961).

28. Rath, R., Das, R., Mishra, G., Mohapatra, B. & Ramakrishna, C. Bancroftian filariasis in two selected rural communities in Puri District: Orissa—a comparative study of filariometric data. *J. Commun. Dis.* **16**, 104-112 (1984).

29. Varma, B., Dass, N. & Sinha, V. Filariasis in the Rural Population around Bhagalpur Town. Parts I. *Indian J. Malariol.* **15**, 285-292 (1961).

30. Chand, D., Singh, M., Gupta, B. & Srivastava, R. A note on filariasis in Gonda Town (Uttar Pradesh). *Indian J. Malariol.* **15**, 39-17 (1961).

31. Rajagopalan, P., Kazmi, S. & Mani, T. Some aspects of transmission of Wuchereria bancrofti and ecology of the vector Culex pipiens fatigans in Pondicherry. *Indian J. Med. Res.* **66**, 200-215 (1977).

32. Chand, D., Singh, M. & WAS, L. Filariasis in Gorakhpur District, Uttar Pradesh. *Indian J. Malariol.* **16**, 269-276 (1962).

33. Jain, D., Ravindranathan, T., Ghosh, T., PILLAI, K. & Rao, C. Prophylactic effect of diethylcarbamazine on W. bancrofti filariasis. *J. Commun. Dis.* **19**, 317-321 (1987).

34. Dondero, T. J.,Jr *et al*. Clinical manifestations of Bancroftian filariasis in a suburb of Calcutta, India. *Am. J. Trop. Med. Hyg.* **25**, 64-73 (1976).
